# Case of Omental Hernia

**Published:** 1845-12-01

**Authors:** Alden S. Sprague


### Case of Operation for Omental Hernia,

*With removal of Spermatic Cord and Testicle.*

Communicated for the Buffalo Medical Journal, by ALDEN S. SPRAGUE, M. D.

The following case occurred several years ago: A gentleman in Royalton, Niagara Co., had been afflicted with hernia for 20 years. I was called to visit him after he had suffered from strangulation for five days. The hernial protrusion was direct, inguinal, and omental; the symptoms being those usually attending cases of that kind, but not in a very aggravated degree. He had been seen by several medical men previous to my being called, all of whom had successively attempted to reduce the hernial tumor by taxis, but without success. I therefore deemed it not prudent to delay an operation which then seemed the only alternative. Previously to its commencement, however, I bled the patient to faintness, and, in the meantime, made some efforts to effect a reduction, but with no better success than my predecessors. The patient and his friends were now extremely anxious that the operation should be resorted to, and he was accordingly placed on the table, after the immediate danger, and the small chance of recovery had been pointed out to them.

The incision was made in the usual manner, and carried down to the sac. This was greatly thickened, and its omental contents found to adhere extensively to the spermatic cord. A considerable time was spent in endeavoring to separate the diseased mass from the cord, but it was found to be utterly impracticable. The state of things at this time appeared rather embarrassing. I decided, however, to remove not only the sac and its contents, but the spermatic cord and testicle also. Accordingly I commenced an incision as near to the abdominal ring as practicable, and divided as much of the omentum as possible, tying two or three small arteries. I then found the spermatic artery and secured it by ligature; after which I completed my first incision. The divided mass, accompanied by the spermatic cord and testicle, was next removed. The testicle was much enlarged, softened, and surrounded by a considerable quantity of semi-transparent, gelatinous fluid. Adhesions had taken place at the ring, which were broken up with the handle of the scalpel and the finger nail. The division of the stricture was made directly inward. The parts were then returned into the abdomen without difficulty, and the wound dresed in the usual manner. A dose of oil was shortly exhibited, which operated well. He was left in the charge of a young medical gentleman who had assisted in the operation, and recovered without any unpleasant symptoms. The mass removed weighed a trifle less than eight ounces.

BUFFALO, October, 1845.

